# Epstein-Barr virus-encoded EBNA1 inhibits the canonical NF-κB pathway in carcinoma cells by inhibiting IKK phosphorylation

**DOI:** 10.1186/1476-4598-9-1

**Published:** 2010-01-05

**Authors:** Robert Valentine, Christopher W Dawson, Chunfang Hu, Khilan M Shah, Thomas J Owen, Kathryn L Date, Sonia P Maia, Jianyong Shao, John R Arrand, Lawrence S Young, John D O'Neil

**Affiliations:** 1 Cancer Research UK Cancer Centre, School of Cancer Sciences, University of Birmingham, Vincent Drive, Edgbaston, Birmingham, B15 2TT, UK; 2 Department of Virology, Faculty of Medicine, Imperial College London, St. Mary's Campus, Norfolk Place, London, W2 1PG, UK; 3 Research Funding, Science Operations and Funding Directorate, Cancer Research UK, 61 Lincoln's Inn Fields, PO Box 123, London WC2A 3PX, UK; 4 Dept. of Pathology, Sun Yat-Sen University Cancer Center, 651 Dong Feng Road East, Guangzhou, 510060, China

## Abstract

**Background:**

The Epstein-Barr virus (EBV)-encoded EBNA1 protein is expressed in all EBV-associated tumours, including undifferentiated nasopharyngeal carcinoma (NPC), where it is indispensable for viral replication, genome maintenance and viral gene expression. EBNA1's transcription factor-like functions also extend to influencing the expression of cellular genes involved in pathways commonly dysregulated during oncogenesis, including elevation of AP-1 activity in NPC cell lines resulting in enhancement of angiogenesis *in vitro*. In this study we sought to extend these observations by examining the role of EBNA1 upon another pathway commonly deregulated during carcinogenesis; namely NF-κB.

**Results:**

In this report we demonstrate that EBNA1 inhibits the canonical NF-κB pathway in carcinoma lines by inhibiting the phosphorylation of IKKα/β. In agreement with this observation we find a reduction in the phosphorylation of IκBα and reduced phosphorylation and nuclear translocation of p65, resulting in a reduction in the amount of p65 in nuclear NF-κB complexes. Similar effects were also found in carcinoma lines infected with recombinant EBV and in the EBV-positive NPC-derived cell line C666-1. Inhibition of NF-κB was dependent upon regions of EBNA1 essential for gene transactivation whilst the interaction with the deubiquitinating enzyme, USP7, was entirely dispensable. Furthermore, in agreement with EBNA1 inhibiting p65 NF-κB we demonstrate that p65 was exclusively cytoplasmic in 11 out of 11 NPC tumours studied.

**Conclusions:**

Inhibition of p65 NF-κB in murine and human epidermis results in tissue hyperplasia and the development of squamous cell carcinoma. In line with this, p65 knockout fibroblasts have a transformed phenotype. Inhibition of p65 NF-κB by EBNA1 may therefore contribute to the development of NPC by inducing tissue hyperplasia. Furthermore, inhibition of NF-κB is employed by viruses as an immune evasion strategy which is also closely linked to oncogenesis during persistent viral infection. Our findings therefore further implicate EBNA1 in playing an important role in the pathogenesis of NPC.

## Background

Epstein-Barr virus (EBV) is a ubiquitous human γ-herpesvirus that is associated with both lymphoid and epithelial tumours [1], including undifferentiated NPC where there is a near 100% association with EBV infection. Whilst the pattern of EBV latent protein expression varies in different tumour types the EBV nuclear antigen, Epstein-Barr nuclear antigen-1 (EBNA1), is expressed in all EBV-associated malignancies due to its indispensable role in the maintenance and replication of the EBV genome via sequence-specific binding to the viral origin of replication, oriP [2]. Furthermore, as a DNA binding protein EBNA1 interacts with viral gene promoters, thereby contributing to the transcriptional regulation of the EBNAs and of latent membrane protein 1 (LMP1) [3].

In addition to EBNA1's functions that depend on its binding to viral DNA, EBNA1 can also interact with host cell proteins, including the ubiquitin-specific protease USP7 which has been implicated in the destabilisation of p53 by binding with a higher affinity to the same region of USP7 as do p53 and MDM2. This suggests that EBNA1 can protect against either UV- or p53-induced apoptosis [4]. Whilst a more direct involvement of EBNA1 in carcinogenesis has been suggested by the ability of B-cell-directed EBNA1 expression to produce B-cell lymphomas in transgenic mice [5], other data are not supportive of such a role [6]. Thus, studies using dominant-negative EBNA1 in an LCL with an integrated EBV genome revealed that EBNA1 had no effect on cell growth or cellular gene expression [7] whilst other work in which EBNA1 was expressed in Akata BL cells previously cleared of EBV infection demonstrated that EBNA1 expression alone is not sufficient to confer tumourigenic potential [8,9]. However, in support of a role for EBNA1 in carcinogenesis we and others have demonstrated that EBNA1's transcription factor-like functions are not confined to the regulation of viral genes but also extend to the regulation of host cell gene expression. This has been demonstrated in the context of B-cells where EBNA1 has been shown to induce the expression of CD25, RAG1, RAG2 and CCL20 [10-12] whilst in epithelial cells we have established that expression of EBNA1 results in the differential regulation of cellular genes involved in translation, transcription and cell signalling [13,14]. We have documented that EBNA1 enhances STAT1 expression which sensitises cells to interferon-induced STAT1 activation, modulates signalling in the TGFβ1 pathway, and increases AP-1 activity resulting in the enhancement of host cell mechanisms involved in angiogenesis and metastasis [13,14]. The mechanism whereby EBNA1 enhances AP-1 activity was determined to be via EBNA1 binding to the promoters of the AP-1 subunits c-Jun and ATF2 [13]. Furthermore, potential EBNA1 binding sites have been found in the promoters of numerous other cellular genes [15].

An *in silico *promoter analysis of gene expression microarray data from EBNA1-expressing carcinoma cells revealed that 15% (362 out of 2454) of the promoters of cellular genes differentially regulated by EBNA1 contained NF-κB DNA binding motifs [13,14] (and unpublished data). It is well established that the EBV-encoded LMP1 activates the NF-κB cascade [3] and that the EBV-encoded latent membrane protein 2A (LMP2A) inhibits NF-κB activity in carcinoma cell models [16]. However, the pattern of expression of these viral proteins varies in NPC biopsies whilst EBNA1 is always expressed due to its key role in EBV genome maintenance. Furthermore, dysregulation of the NF-κB pathway has been implicated and documented in the pathogenesis of a wide range of cancers [17]. These observations coupled with reports that the functional homologues of EBNA1 (LANA and ORF73, encoded by KSHV and MuHV-4 respectively) inhibit NF-κB activity [18,19] prompted us to investigate whether EBNA1 also influences NF-κB activity in carcinoma cells and if this may contribute to the development of EBV-associated epithelial cell tumours such as nasopharyngeal carcinoma (NPC).

## Methods

### Cell lines and tissue culture

Ad/AH (a human adenocarcinoma cell line derived from the nasopharynx), Hone1 (an EBV-negative NPC cell line), AGS (a human gastric-carcinoma derived cell line) and derivatives stably expressing EBNA1 at levels comparable to those found in EBV infection, Ad/AH cells stably infected with a recombinant EBV and C666-1 (an EBV-positive cell line derived from an undifferentiated EBV-positive NPC) were cultured as previously described [13,14]. Neither Ad/AH cells stably infected with EBV or the C666-1 cell line express the EBV-encoded latent membrane protein 1 (LMP1). TNFα and IL-1β (Peprotech, London, UK) were reconstituted in serum-free growth medium to a concentration of 100 ng/ml, and stimulations carried out for 1 hour prior to harvesting.

### Luciferase assays and transient transfections

Dual luciferase reporter assays were performed according to the manufacturer's instructions (Promega, Southampton, UK, cat. no. E1980) with cells cultured as previously described [13]. Cells were transfected with the following plasmids using Lipofectamine (Invitrogen, Renfrew, UK) following the manufacturer's instructions: pSG5-EBNA1 [20], pGL3-basic (Promega), 3 enhancer-ConA (an NF-κB-dependent luciferase reporter construct in which transcription of the firefly luciferase gene is driven by three NF-κB binding sites) [21], dnEBNA1 (M15 EBNA1 dominant-negative mutant [22]), and a control Renilla luciferase plasmid (pRL-TK; Promega). All assays were carried out in biological and technical triplicate and are represented as the mean of three independent experiments.

### Construction of wild-type and mutant EBNA1 lentivirus vectors

DNA encoding wild-type EBNA1 was excised from pSG5-EBNA1 [20] using EcoRI and BsaHI and end-filled using Klenow DNA polymerase. Adenine nucleotides were added using *Taq *DNA polymerase and the resulting DNA was ligated into pCR8 (Invitrogen, cat. no. K2500-20), following the manufacturer's instructions. DNA encoding the dGA, dnEBNA1 (d395-450), d8-67, d41-376, d61-83 and d325-376 EBNA1 mutants [23] was amplified by PCR using the following primers specific to the flanking vector sequences; 5'GCCGGATCCCCCACTGCTTACTGGCTTAT-3' and 5'-GCCGTCGACGGCAAACAACAGATGGCTGGCAA-3'. These were inserted into pCR8 following the manufacturer's instructions. An empty vector control (Vector) was generated by self ligation of pCR8. The vectors derived above were recombined with pLenti6/R4R2/V5-DEST (Invitrogen, cat. no. K591-10 and K5910-00) and pENTR5 containing the human metallothionein II promoter, following the manufacturer's instructions. In the transient transfection experiments presented here zinc stimulation was not required as the metallothionein II promoter was found to exhibit inherent leakiness (data not shown).

### Electrophoretic mobility shift assay (EMSA)

Nuclear extracts were prepared according to the manufacturer's instructions (Pierce Biotechnology, Illionois, USA, cat. no. 78833) and EMSA analysis was carried out on 5 μg of nuclear protein according to the manufacturer's instructions (LI-COR Biosciences, Cambridge, UK, doc. 982-07487) using an NF-κB consensus probe (sense oligonucleotide 5'-AGTTGAGGGGACTTTCCCAGGC-3') which was either 5' IRDye700 labelled or unlabelled for cold competition. EMSA gels were analysed and images were captured and quantified using the LI-COR Odyssey infrared laser imaging system. EMSAs were repeated for three independent biological replicates.

### TransAM analysis

Nuclear protein extracts were isolated according to manufacturer's instructions (Active Motif, Rixensart, Belgium, cat. no. 40010). The NF-κB subunits p65 and p50 present in active dimers were measured using the ELISA based TransAM NF-κB family kit (Active Motif, cat. no. 43296) according to the manufacturer's instruction. Data are presented relative to the supplied internal Raji cell lysate control and are represented as the mean of three independent experiments.

### RT-PCR, immunoblotting, immunofluorescence and immunohistochemistry

RNA was extracted using EZ-RNA total RNA isolation kit (Geneflow, Staffordshire, UK) and was reverse transcribed for RT-PCR with Superscript III (Invitrogen), following the manufacturer's instruction. RT-PCR was carried out using standard procedures with the primers listed in Table 1. Standard immunoblotting procedures [14] were used to detect proteins using the antibodies listed in Table 2. All assays were carried out in biological and technical triplicate and are represented as the mean of three independent experiments. Tissue arrays were constructed and assayed at the Department of Pathology, Cancer Centre, Sun Yat-Sen University (Guangzhou, Guangdong, China) as follows; formalin-fixed paraffin-embedded blocks were obtained from the archives of the Sun Yat-Sen University pathology department. The matching H&E-stained slides were reviewed and screened, and samples containing both NPC tumour and adjacent nasopharyngeal mucosae were chosen for tissue array construction. Each case was represented by a mean of 4 cores with 2 tumours and 2 normal nasopharyngeal mucosa using a 0.6 mm punch. Immunohistochemistry was performed using the agitated low temperature epitope retrieval (ALTER) method [24]. Immunohistochemical staining was carried out for p65 and sections were counterstained with haematoxylin.

**Table 1:** Oligonucleotide primers used in RT-PCR

Gene	RT-PCR primer oligonucleotides (5'-3')
TNFR1	Forward: GCTCCTTCACCGCTTCAGA Reverse: CCAATGAAGAGGAGGGATAAA

TNFR2	Forward: CAGCCTTGGGTCTACTAATA Reverse: GCCACCAGGGGAAGAATC

IL1R1	Forward: GTGATGAATGTGGCTGAAA Reverse: CTGGGTCATCTTCATCAAT

IL1R2	Forward: CAGAGTTTTTGAGAATACAGAT Reverse: GTCCCCCTCACACTTAGAA

C/EBPβ (NF-IL6)	Forward: GACTTCCTCTCCGACCTCT Reverse: TGCTTGTCCACGGTCTTCTT

IL1α	Forward: GAAGAAGAGACGGTTGAGTTT Reverse: GCACTGGTTGGTCTTCATCT

A20	Forward: CCCAGACCACACAAGGCA Reverse: GGCAGTATCCTTCAAACAT

EBNA1	Forward: CCGCAGATGACCCAGGAGAA Reverse: TGGAAACCAGGGAGGCAAAT

GAPDH	Forward: GCCTCCTGCACCACCAACTG Reverse: CGACGCCTGCTTCACCACCTTCT

**Table 2:** Antibodies used in immunoblotting (IM), immunofluorescence (IF) and immunohistochemistry (IHC)

Protein	Primary antibody	Species	Application
β-actin	Sigma-Aldrich (A5441)	Mouse	IM

EBNA1	A.M.	Human sera	IM

EBNA1	R4	Rabbit	IF

IKKα	Santa Cruz (sc-7607)	Mouse	IM

IKKβ	Cell signalling (L570)	Rabbit	IM

IKKγ	Santa Cruz (sc-8330)	Rabbit	IM

IκBα	Cell signalling (9242)	Rabbit	IM

p65/RelA	Cell signalling (3034)	Rabbit	IM

p65/RelA	Santa Cruz (sc-372)	Rabbit	IF, IHC

Phospho-IκBα	(Ser32) Cell signalling (2859)	Rabbit	IM

Phospho-IKKα/β	(Ser176/180) Cell signalling (2687)	Rabbit	IM

Phospho-p65	(Ser536) Cell signalling (3031)	Rabbit	IM

### Statistics

Where appropriate, statistical significance was calculated by performing a Sudent's t-test having first determined equal or unequal variance by using an F-test.

## Results

### EBNA1 represses p65 NF-κB activity in carcinoma cells

**Figure 1:**
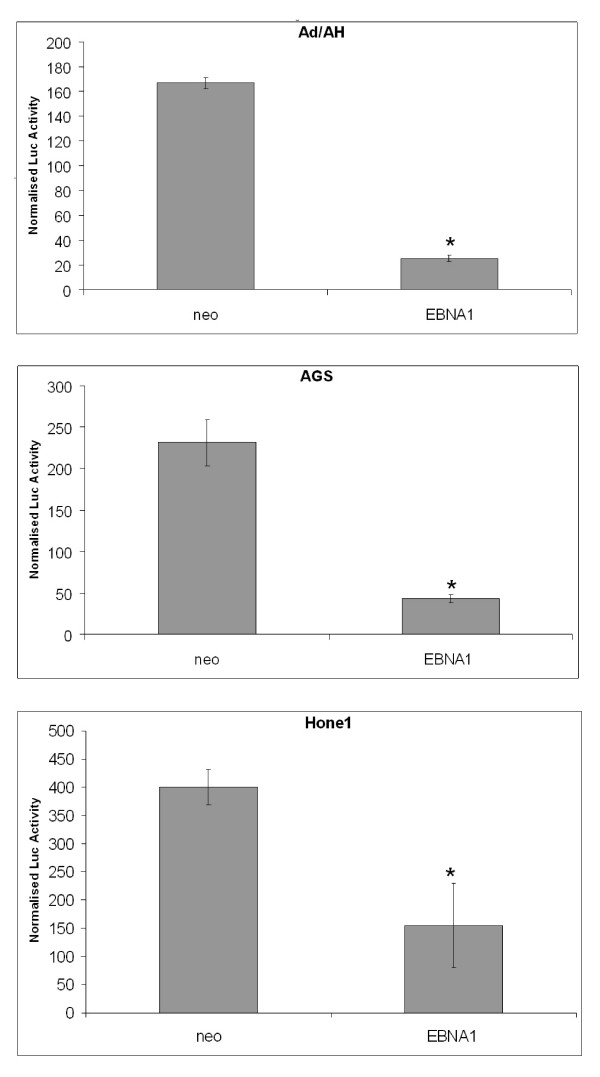
**EBNA1 inhibits NF-κB luciferase reporter activity in Ad/AH (upper), AGS (middle) and Hone1 (lower) cell lines stably expressing either EBNA1 or a neomycin control plasmid (neo)**. Reporter assays were performed in biological and technical triplicate and error bars indicate SD (* = P < 0.05 relative to EBNA1-free controls).

**Figure 2:**
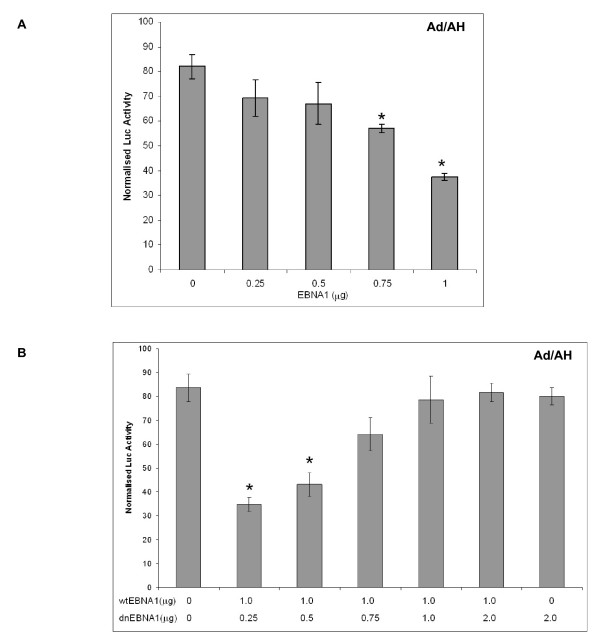
**(A) Transient transfection of increasing concentrations of the EBNA1 expression plasmid pSG5-EBNA1 into parental Ad/AH cells inhibits NF-κB luciferase reporter activity in a dose-dependent manner**. (B) Transient transfection of increasing concentrations of a dominant-negative EBNA1 (dnEBNA1) abrogates the ability of wild-type EBNA1 (wtEBNA1) to inhibit NF-κB luciferase activity in parental Ad/AH cells. Reporter assays were performed in biological and technical triplicate and error bars indicate SD (* = P < 0.05 relative to EBNA1-free controls).

**Figure 3:**
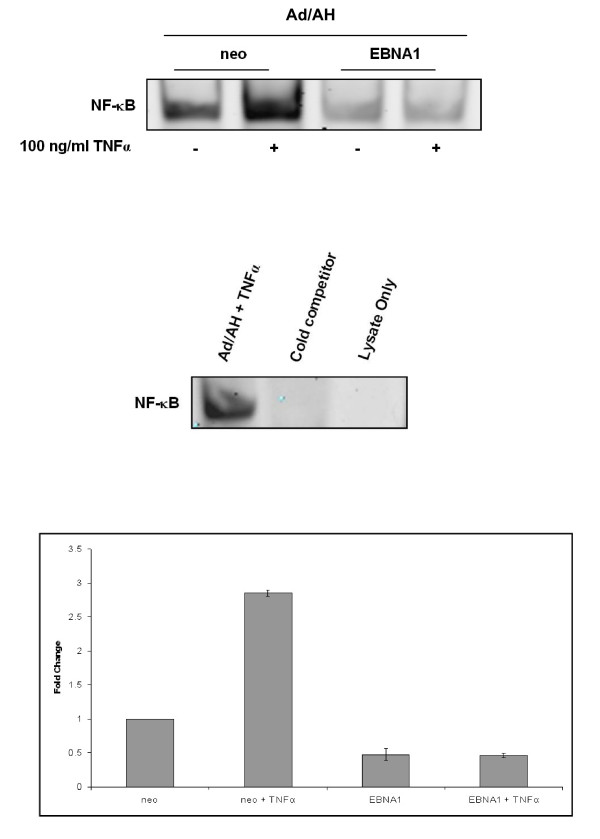
**EMSA analysis with quantitative densitometry (lower panel) demonstrating that stable EBNA1 expression in Ad/AH cells inhibits basal NF-κB DNA binding and in response to TNFα, relative to Ad/AH cells expressing a neomycin control plasmid (neo) (upper)**. Cold competition using a 100-fold excess of unlabelled NF-κB probe (cold competitor) ablates NF-κB binding (middle). EMSAs were performed in triplicate.

**Figure 4:**
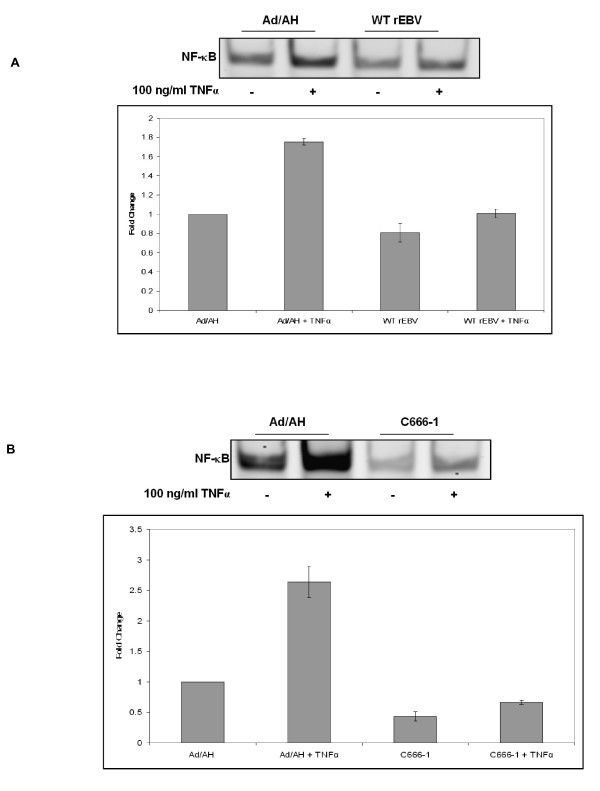
**EMSA analysis with quantitative densitometry demonstrating that (A) Ad/AH cells stably infected with rEBV exhibit reduced NF-κB DNA binding, both basally and in response to TNFα, relative to Ad/AH parental cells**. (B) C666-1 cells (EBV-positive) exhibit reduced NF-κB DNA binding, both basally and in response to TNFα, relative to Ad/AH parental cells. EMSAs were performed in triplicate.

To assess whether EBNA1 influenced NF-κB activity we initially performed luciferase reporter assays in a range of carcinoma cell lines using a synthetic NF-κB reporter and found that NF-κB activity in Ad/AH, AGS and Hone1 cells stably expressing levels of EBNA1 comparable to those found in EBV infection was inhibited by 8, 5 and 2.6 fold, respectively (Fig. 1). Transient expression of EBNA1 in Ad/AH cells achieved by transfection using a range of concentrations of EBNA1 plasmid DNA resulted in a dose-dependent decrease in NF-κB reporter activity with a 2-fold reduction seen at the highest concentration of input DNA (Fig. 2A). To assess whether inhibition of NF-κB required a fully functional EBNA1, a dominant-negative EBNA1 (dnEBNA1) was titrated against wild-type EBNA1 in Ad/AH cells. Increasing doses of dnEBNA1, which dimerises with wild-type EBNA1 impairing its function, resulted in almost complete abrogation of the ability of wild-type EBNA1 to inhibit NF-κB activity (Fig. 2B). In addition, the dnEBNA1 alone had no effect on NF-κB reporter activity (Fig. 2B). Next we performed electromobility shift assays (EMSA) using a consensus NF-κB probe to assess whether the reduction in reporter activity was due to a reduction in NF-κB DNA binding. EMSAs performed on Ad/AH cells stably expressing EBNA1 indicated a 2-fold basal reduction in band intensity indicating a reduction in nuclear protein binding to the NF-κB probe, when compared with the neo control cells, which was consistent with our observed reduction in reporter activity (Fig. 3). Furthermore, the ability of two different pro-inflammatory cytokines, TNFα and IL-1β, to enhance NF-κB DNA binding was ablated in those cells stably expressing EBNA1 (Fig. 3 and data not shown). Similar results were observed in Ad/AH cells stably infected with a recombinant EBV (rEBV) carrying the neomycin drug selectable marker (Fig. 4A). In addition, the ability of TNFα, a potent activator of the canonical NF-κB pathway, to enhance binding with the NF-κB probe in C666-1 cells was considerably lower than in Ad/AH parental cells (Fig. 4B). EMSAs performed on Ad/AH cells stimulated with TNFα incubated with both the labeled NF-κB probe and a 100-fold excess of unlabeled probe (cold competition) resulted in complete abrogation of probe binding (Fig. 3, middle panel), thus demonstrating the high degree of specificity in the EMSA assays.

**Figure 5:**
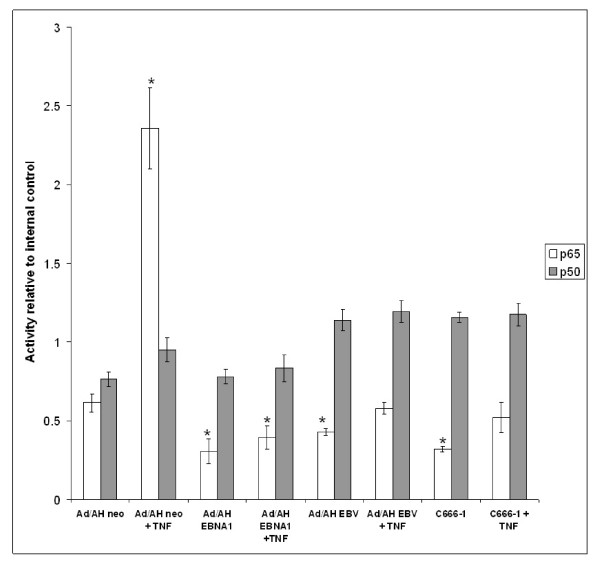
**TransAM analysis demonstrating the p65 and p50 composition of transcriptionally competent NF-κB dimers present in Ad/AH cells stably expressing EBNA1 or a neomycin control vector (neo), Ad/AH cells stably infected with rEBV and C666-1 cells, under basal conditions and following stimulation with 100 ng/ml TNFα**. TransAM analysis was performed in biological and technical triplicate and error bars indicate SD (* = P < 0.05 relative to Ad/AH Neo control).

Having demonstrated that general NF-κB activity and NF-κB DNA binding in EBNA1 expressing cells was reduced, we sought to determine the relative abundance of the canonical NF-κB subunits p65 and p50 in active nuclear complexes bound to target DNA. We chose to study p65 and p50 because heterodimers of these NF-κB subunits are the most abundant form of NF-κB and exhibit the most powerful transcriptional activation potential [25]. Furthermore, the canonical NF-κB pathway is most commonly associated with innate immunity in general and with cellular differentiation in epithelial cells, both of which impact upon the pathogenesis of NPC. TransAM analysis in Ad/AH cells demonstrated a 4-fold enrichment of p65-containing dimers in neo control cells stimulated with TNFα (relative to un-stimulated neo cells), whereas the abundance of p50 did not change significantly (Fig. 5). In contrast, there was a 2-fold reduction in the basal amount of p65-containing dimers in Ad/AH cells stably expressing EBNA1, when compared with the un-stimulated neo control, and these cells were refractory to stimulation with TNFα. However, the amount of p50-containing dimers in Ad/AH cells stably expressing EBNA1 both pre and post TNFα stimulation was not significantly different from the levels observed in the neo control cells. Results similar to those found in Ad/AH cells stably expressing EBNA1 were also observed in Ad/AH cells stably infected with rEBV and in C666-1 cells. Interestingly, the amount of p50-containing dimers was marginally increased in Ad/AH cells stably infected with rEBV and in the C666-1 cells and this was unaffected by stimulation with TNFα.

### EBNA1 inhibits the phosphorylation and nuclear translocation of p65 in carcinoma cells

**Figure 6:**
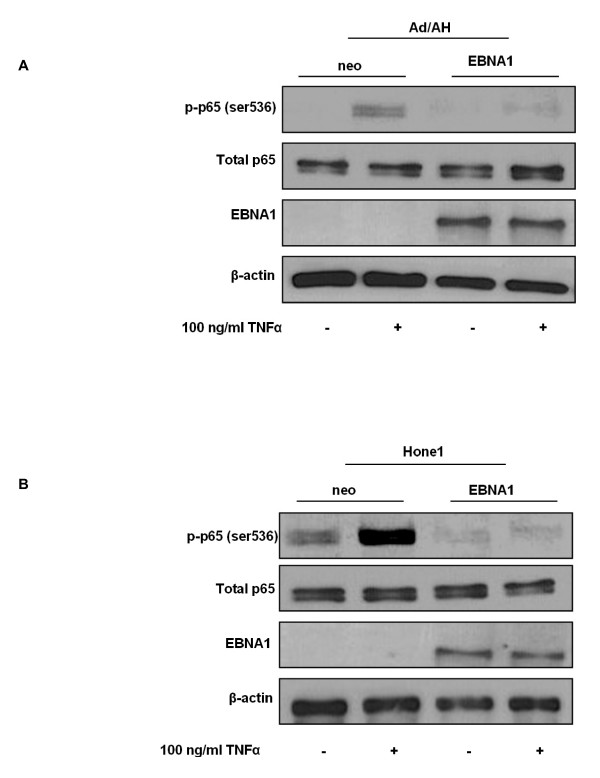
**EBNA1 inhibits p65 phosphorylation in carcinoma cell lines**. Western blot analyses of total and phosphorylated (ser 536) p65 in (A) Ad/AH and (B) Hone1 cells stably expressing EBNA1 or a neomycin control vector (neo) under basal conditions or following stimulation with TNFα. Western blotting for EBNA1 and β-actin serve as EBNA1 expression and protein loading controls, respectively.

**Figure 7:**
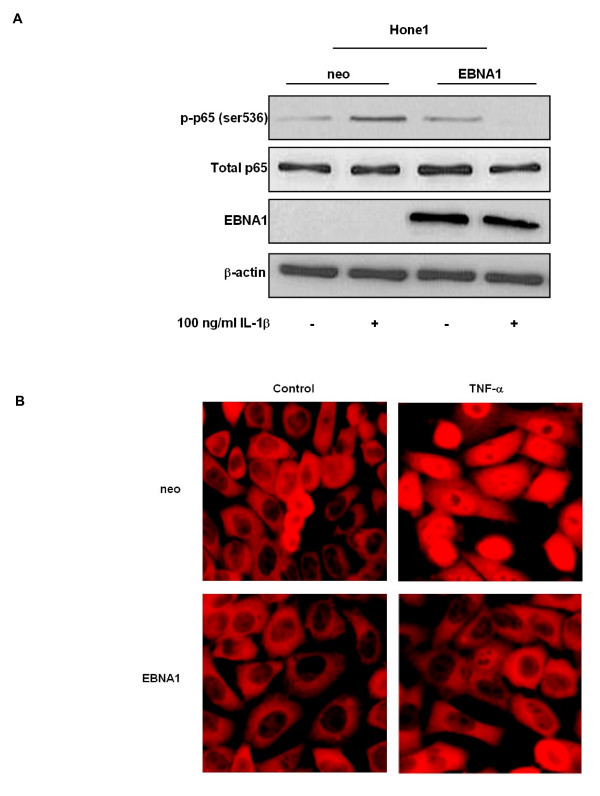
**EBNA1 inhibits p65 phosphorylation and nuclear translocation in carcinoma cell lines**. Western blot analyses of total and phosphorylated (ser 536) p65 in (A) Hone1 cells stably expressing EBNA1 or a neomycin control vector (neo) under basal conditions or following stimulation with IL-1β. Western blotting for EBNA1 and β-actin serve as EBNA1 expression and protein loading controls, respectively. (B) Immunofluorescent staining for p65 in Ad/AH cells stably expressing EBNA1 or a neomycin control vector (neo) under basal conditions or following stimulation with 100 ng/ml TNFα.

We next sought to determine whether the EBNA1-induced reduction in NF-κB activity and reduced levels of p65 in active NF-κB dimers was as a result of alterations in the expression or phosphorylation status of p65. In its transcriptionally inactive form unphosphorylated p65 NF-κB is retained in the cytoplasm via interactions with specific inhibitors, the IκBs. Upon stimulation of the canonical NF-κB pathway the inhibitory IκBα protein becomes phosphorylated, ubiquitinated and degraded by the 26S proteasome. NF-κB dimers subsequently translocate to the nucleus where phosphorylated p65-containing dimers can modulate the expression of target genes [26]. Specifically, phosphorylation of p65 at Ser 536 by IKKα and IKKβ has been implicated in p65 nuclear translocation and transcriptional activity [26]. Immunoblot analysis confirmed enhanced serine 536 phosphorylation of p65 following TNFα stimulation of Ad/AH and Hone1 neo control cells as expected (Fig. 6A and 6B, respectively). In contrast, there was almost complete inhibition of p65 phosphorylation in stable EBNA1-expressing Ad/AH and Hone1 cells basally and following TNFα stimulation. Furthermore, the reduction in p65 phosphorylation in EBNA1-expressing cells was not due to a reduction in total levels of p65 protein (Fig. 6A and 6B, respectively). EBNA1-expressing Hone1 cells were also refractory to stimulation with another potent activator of the canonical NF-κB pathway, IL-1β, exhibiting reduced p65 phosphorylation in EBNA1-expressing cells which was in contrast to enhanced levels of phospho-p65 observed in the neo control cells (Fig. 7A). Immunofluorescence staining in Ad/AH cells also revealed that the ability of TNFα to stimulate translocation of p65 from the cytoplasm to the nucleus, as seen in the neo control cells, was almost completely inhibited in cells stably expressing EBNA1 (Fig. 7B). These observations were, therefore, in agreement with the above reporter assays, EMSA and TransAM data.

### EBNA1 inhibits the phosphorylation of IκBα and IKKα/β in carcinoma cells

The activation of the canonical NF-κB pathway is tightly regulated by signals such as pro-inflammatory cytokines, that stimulate the IκB kinase complex (IKK) to phosphorylate the inhibitory IκBs which marks them for ubiquitin-mediated degradation. This allows free NF-κB to translocate to the nucleus where it activates target genes. The IKK complex is composed of two catalytic subunits, IKKα and IKKβ, which contain N- terminal serine/threonine kinase domains and a regulatory subunit, IKKγ (NEMO), that does not exhibit kinase activity but is essential for IKK phosphorylation and activation of upstream kinases [27]. Whilst both IKKα and IKKβ cooperate for IκB phosphorylation, IKKβ is indispensable for signalling via the canonical NF-κB pathway [28].

**Figure 8:**
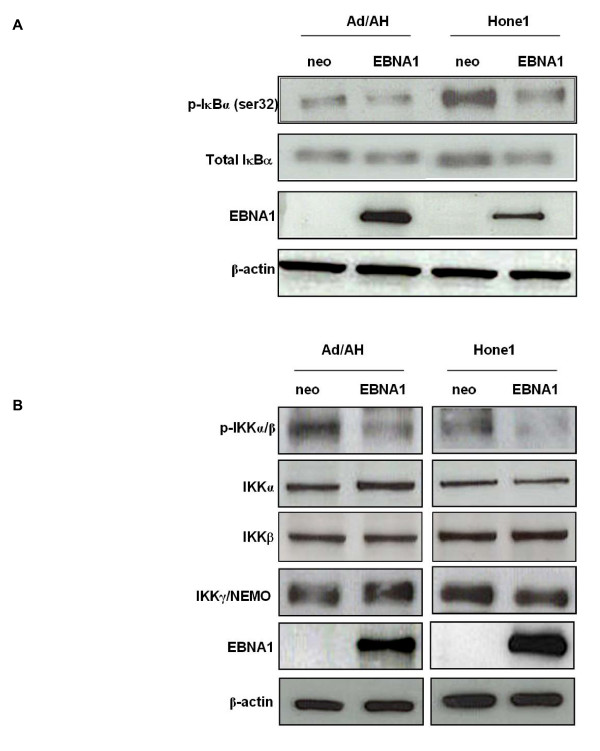
**EBNA1 inhibits the phosphorylation of IκBα and IKKα/β in carcinoma cells**. (A) Western blot analysis of total and phosphorylated IκBα (ser32) in Ad/AH and Hone1 cells stably expressing EBNA1 or a neomycin control vector (neo).(B) Western blot analysis of total IKKα, IKKβ, IKKγ and phosphorylated IKKα/β (IKKα Ser176/180 and IKKβ Ser177/181) in Ad/AH and Hone1 cells stably expressing EBNA1 or a neomycin control vector (neo). Western blotting for EBNA1 and β-actin serve as EBNA1 expression and protein loading controls, respectively.

Having demonstrated that stable EBNA1 expression results in reduced levels of phospho-p65 and its translocation to the nucleus we sought to determine whether EBNA1 achieved this by affecting the expression and/or phosphorylation status of the NF-κB inhibitory subunit IκBα. Immunoblotting demonstrated that stable EBNA1 expression in Ad/AH and Hone1 cells resulted in a decrease in phosphorylation of IκBα at ser32, relative to the neo control cells (Fig. 8A). In contrast, stable EBNA1 expression did not alter the expression of total IκBα in the Ad/AH cells. In the Hone1 cells stable EBNA1 expression resulted in a marginal reduction in the expression of total IκBα, which is itself an NF-κB regulated gene.

We then performed immunoblotting to determine whether EBNA1 achieved a reduction in IκBα and p65 phosphorylation by affecting components of the IKK complex. There was a marked reduction in the levels of serine phosphorylation within the activation loops of both IKKα/β, a prerequisite for activation, in Ad/AH and Hone1 cells stably expressing EBNA1, when compared with the neo control cell lines (Fig. 8B). In contrast, there was no appreciable difference in the total levels of IKKα, IKKβ or IKKγ (Fig. 8B) which demonstrated that the reduction in phospho-IKKα/β in EBNA1 expressing Ad/AH and Hone1 cells was not due to a reduction in the expression of these catalytic subunits.

### Deletion of EBNA1 domains required for transactivation of EBV encoded genes abrogates the ability of EBNA1 to inhibit NF-κB activity

**Figure 9:**
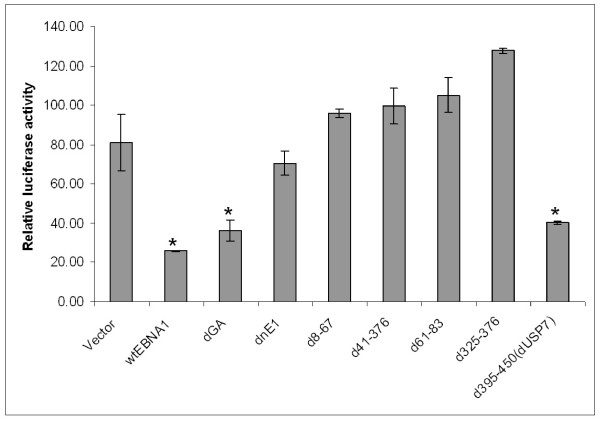
**NF-κB luciferase reporter activity following transient transfection of Ad/AH cells with concentrations of lentivirus vectors that result in equal levels of expression of wild-type EBNA1 and mutants**. Wild-type (wtEBNA1), gly-ala repeat-deleted EBNA1 (dGA) and EBNA1 deleted for the cellular USP7 binding site (d395-450 dUSP7) result in significant inhibition of NF-κB reporter activity, relative to the empty vector control (Vector). In contrast, deletion of EBNA1 domains (d8-67, d41-376, d61-83 and d325-376) characterised as being essential for the transactivation of EBV genes does not result in inhibition of NF-κB reporter activity. Reporter assays were performed in biological and technical triplicate and error bars indicate SD (* = P < 0.05 relative to empty vector control).

Having demonstrated that EBNA1 inhibited the phosphorylation of IKKα/β we asked whether specific domains of EBNA1 were responsible for this phenomenon. Therefore, we cloned wild-type EBNA1 and a selection of EBNA1 domain mutants [23] into a lentiviral expression vector. Amounts of DNA found to yield equal levels of protein expression (data not shown) were transiently transfected into Ad/AH cells and NF-κB luciferase reporter assays were performed (Fig. 9). Transfection of wild-type EBNA1 (wtEBNA1) and EBNA1 lacking the gly-ala repeat region (dGA), from which all subsequent mutants were derived [23], resulted in a significant reduction in NF-κB activity, relative to the empty vector control (Vector), in agreement with our data presented in Fig. 2A. Transfection of a dominant-negative EBNA1 (dnEBNA1) carrying the same DNA sequence as that used in Fig. 2B did not result in a significant reduction in NF-κB reporter activity. Transfection of EBNA1 mutants d8-67, d41-376, d61-83 and d325-376 did not result in a significant reduction in NF-κB reporter activity. Thus deletion of domains of EBNA1 essential to its ability to transactivate viral gene expression ablated its ability to inhibit NF-κB activity. In contrast, transfection of an EBNA1 mutant deleted for the binding site of the deubiquitinylating enzyme USP7 [4] (d395-450) resulted in significant inhibition of NF-κB reporter activity. Therefore deletion of the USP7 binding domain had not altered the ability of EBNA1 to inhibit NF-κB activity. These data therefore suggested that the mechanism by which EBNA1 was able to inhibit phosphorylation of IKKα/β was likely to be through EBNA1s ability to modulate the expression of cellular genes. Therefore we sought to determine whether EBNA1 influenced the expression of a selection of genes reported to regulate IKK activity and the canonical NF-κB pathway in general.

### EBNA1 does not alter the expression of IL-1 or TNF receptors in carcinoma cells

**Figure 10:**
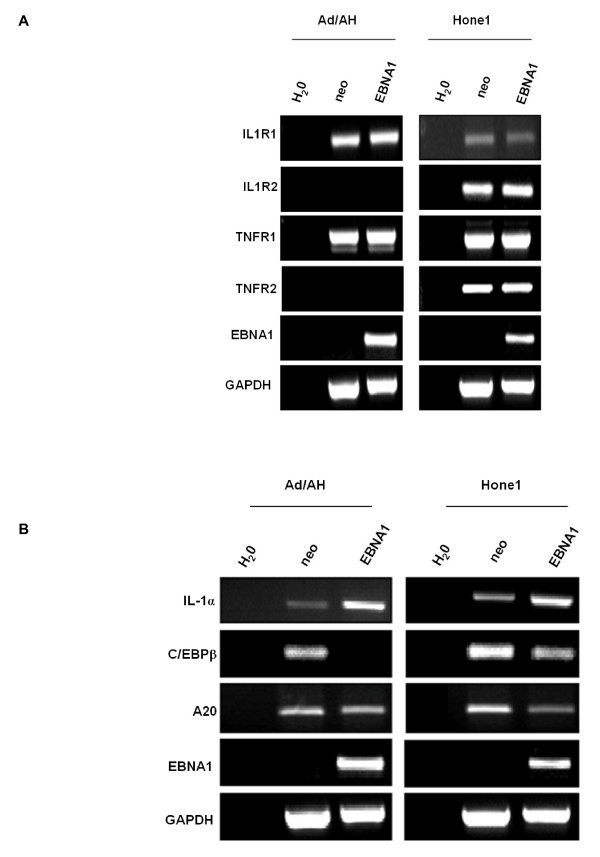
**RT-PCR analysis for (A) the interleukin-1 receptors 1 and 2 (IL1R1 and IL1R2), TNF receptors 1 and 2 (TNFR1 and TNFR2) in Ad/AH and Hone1 cells stably expressing EBNA1 or a neomycin control vector (neo) indicate that EBNA1 does not alter the natural level of expression of IL-1 or TNF receptors in carcinoma cells**. (B) RT-PCR analysis for IL-1α, C/EBPβ and A20 in Ad/AH and Hone1 cells stably expressing EBNA1 or a neomycin control vector (neo) indicate that EBNA1 does not inhibit NF-κB activity through down-regulation of pro-inflammatory cytokines or up-regulation of C/EBPβ or A20. RT-PCR for EBNA1 and GAPDH serve as EBNA1 and loading controls, respectively, whilst the water only sample (H_2_O) serves as a general PCR control.

As IL-1 and TNF activate the canonical NF-κB pathway by binding with their cognate receptors we next examined whether the EBNA1-induced reduction in IKKα/β phosphorylation was as a consequence of EBNA1 modulating the expression of IL-1 and/or TNF receptors. RT-PCR analysis revealed that EBNA1 did not alter the natural levels of expression of the IL-1 receptors 1 and 2 (IL1R1 and IL1R2) or TNF receptors 1 or 2 (TNFR1 and TNFR2) in Ad/AH and Hone1 cells (Fig. 10A). Therefore it is unlikely that EBNA1 modulates NF-κB by influencing the ability of cells to respond to these pro-inflammatory ligands.

### EBNA1 does not inhibit NF-κB activity through down-regulation of pro-inflammatory cytokines or up-regulation of C/EBPβ or A20

We next sought to determine whether EBNA1 inhibited NF-κB activity by down-regulating the expression of IL-1 and TNFα. RT-PCR demonstrated that whilst TNFα and IL-1β expression could not be detected we surprisingly found IL-1α to be up-regulated by EBNA1 in both Ad/AH and Hone1 cells (Fig. 10B and microarray data not shown). This suggested that reduced stimulation of the NF-κB pathway is not responsible for the low levels of NF-κB activity observed in EBNA1 expressing epithelial cells. Intriguingly, however, elevated IL-1α is a marked feature of NPC suggesting that EBNA1 may contribute to this phenotype [29,30].

The transcription factor C/EBPβ has been implicated in NF-κB inhibition by preventing p65 phosphorylation [31]. RT-PCR revealed that EBNA1 expression in the nasopharyngeal cell lines was associated with C/EBPβ down-regulation and was therefore unlikely to be involved in the mechanism by which EBNA1 inhibited NF-κB activity in epithelial cells (Fig. 10B).

The microarray data used for the *in silico *promoter analysis that implicated EBNA1 as having a role in NF-κB modulation reported that expression of A20, which inhibits NF-κB machinery up-stream of the IKK complex, was up-regulated 2.5-fold. RT-PCR analysis, however, indicated that A20 was down-regulated in Ad/AH and Hone1 cells stably expressing EBNA1 and was therefore unlikely to be involved in the mechanism by which EBNA1 inhibited NF-κB activity in epithelial cells (Fig. 10B)

### p65 is localised to the cytoplasm in NPC tumour cells

**Figure 11:**
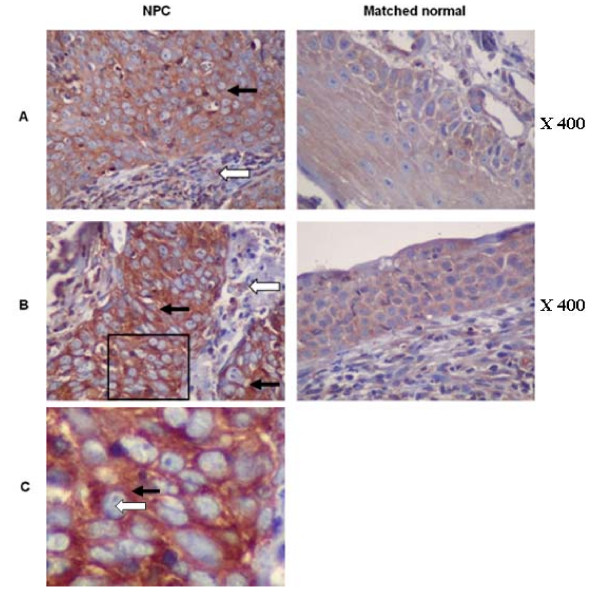
**In the tumour cells of NPC biopsies p65 is localised in the cytoplasm**. Immunohistochemical staining for p65 was carried out on tissue arrays containing 11 NPC biopsies with matched normal nasopharyngeal control tissue isolated from the same patients. Two examples of this staining (A and B) are presented where black arrows indicate tumour cell islands and white arrows indicate the cellular infiltrate surrounding tumour cells. A higher magnification of the cells bounded by a black box in (B) is presented in (C) where the black arrow indicates the cytoplasm and the white arrow indicates the nucleus. Sections were counterstained with haematoxylin.

Previous reports have demonstrated in EBV-positive NPC xenografts and EBV-positive NPC biopsies that p65 is localised to the cytoplasm in tumour cells [32,33]. Having demonstrated that there was a reduction in active nuclear p65 in carcinoma cells stably expressing EBNA1, infected with rEBV and in C666-1 cells we sought to confirm the p65 status in NPC biopsies. Immunohistochemical staining for p65 was carried out on tissue arrays containing 11 NPC biopsies with matched normal nasopharyngeal control tissue. This demonstrated that the level of p65 staining was elevated in the tumour cells of 6 out of 11 NPC biopsies, relative to the matched normal tissue sections, with the p65 staining in the remaining 5 tumour samples being indistinguishable from the controls. However, the p65 staining in all cases was exclusively cytoplasmic with no detectable nuclear staining despite the pro-inflammatory environment characteristic of NPC. The p65 staining in the cellular infiltrate of all NPC biopsies was negative. Two examples are presented in Fig. 11.

### Discussion

NF-κB signalling regulates a variety of major cellular processes, including cell growth, differentiation and apoptosis, and it is therefore not surprising that aberrant NF-κB signalling has been implicated and documented in the pathogenesis of a wide range of cancers. In addition, NF-κB signalling impacts upon both adaptive and innate immunity, the latter being crucial to the ability of host cells to mount effective defences against oncogenic viruses such as EBV [34]. In this study we have demonstrated for the first time that NF-κB activity is repressed by both transient and stable expression of EBNA1 in a number of carcinoma cell lines; confirming that the phenomenon is not merely cell line specific or due to clonal variation. This study therefore reveals that, like its homologues from KSHV and MuHV-4 (LANA and ORF73, respectively) [18,19], EBNA1 also has a role in inhibiting the NF-κB pathway and that this is therefore most likely a conserved function amongst these gammaherpesvirus nuclear proteins.

Whilst the mechanism for ORF73-mediated NF-κB inhibition has been determined to be via poly-ubiquitination and subsequent proteasomal-dependent nuclear degradation of p65, which is dependent upon a SOCs box motif present in ORF73, the mechanism for LANA-mediated NF-κB repression remains unknown, although it may be similar as LANA also contains a SOCs box [19,35]. However, examination of EBNA1 reveals no obvious SOCs motif. In contrast, the findings presented here indicate that EBNA1 inhibits the canonical NF-κB pathway in carcinoma lines by inhibiting phosphorylation of the IKK complex upon which several pro-inflammatory signalling cascades converge, enabling EBNA1 to block NF-κB activation in response to a broad range of stimuli.

Whilst we have not fully elucidated the mechanism by which EBNA1 inhibits IKK phosphorylation our data do indicate that deletion of domains of EBNA1 reported to be essential to its ability to transactivate EBV encoded genes [23] abrogated the ability of EBNA1 to inhibit NF-κB activity, whereas deletion of the domain of EBNA1 known to bind with the cellular deubiquitinylating enzyme USP7 [4] had no effect. These data therefore suggest that the ability of EBNA1 to inhibit IKK phosphorylation is most likely via EBNA1 regulating the expression of a cellular gene(s) involved in this process. Subsequent RT-PCR analysis determined that a reduction in IKKα/β phosphorylation was not likely to be as a result of inhibition of the expression of IL-1 or TNFα receptors or their ligands, or by up-regulation of the NF-κB inhibitors C/EBPβ or A20. Interestingly, RT-PCR analysis indicated that A20 was down regulated in Ad/AH and Hone1 cells stably expressing physiological levels of EBNA1, adding credence to our observations that EBNA1 inhibits NF-κB activity as A20 expression is positively regulated by NF-κB [36]. In addition, preliminary observations suggest that EBNA1 does not bind with or relocalise IKKβ to the nucleus and so it is unlikely that this is the mechanism by which nuclear EBNA1 inhibits the phosphorylation and activity of the IKK complex (Figure S1, Additional file 1). The precise mechanism by which EBNA1 inhibits IKKα/β phosphorylation and NF-κB activity is therefore currently under investigation.

Chronic activation of NF-κB is associated with the development of a number of malignancies. Therefore on face value our observation that EBNA1 inhibits canonical NF-κB would appear counter intuitive with regard to the pathogenesis of NPC. However, within the context of epithelial cells it has been reported that NF-κB activation is growth inhibitory. For example, Seitz *et al*. [37] found that the expression of constitutively active p50 and p65 canonical NF-κB subunits in normal epithelial cells resulted in irreversible cell cycle arrest, whereas Gapuzan *et al*. [38] reported that p65 knockout fibroblasts have a transformed phenotype. In addition, expression of a dominant-negative IκBα "super repressor" in murine and human epidermis led to hyperplasia and the development of squamous cell carcinoma (SCC) [39]. Furthermore, Dajee *et al*. [40] showed in SCC biopsies that p65 staining was predominantly cytosolic.

The ability of EBV-encoded LMP1 to activate both the canonical and non-canonical NF-κB pathway has been the subject of many, mostly *in vitro*, studies. However it is becoming increasingly evident that the ability of LMP1 to activate the NF-κB cascade *in vivo *where other EBV latent genes, including EBNA1, are expressed is not so well defined. Recent studies have gone some way to addressing this question by demonstrating in EBV-positive NPC biopsies and xenografts that p65 is located almost exclusively in the cytoplasm and that this is independent of LMP1 expression [32,33]. In agreement with and expanding upon these data, we have demonstrated that whilst the level of p65 was elevated in 6 out of 11 NPC tumours examined, relative to matched normal control tissue from the same patient, in all cases p65 staining was cytoplasmic. Thornburg *et al*. [33] propose that inhibition of p65 in NPC may protect against growth arrest whilst p50/p50 and p50/BCL3 NF-κB could still maintain the tumourigenic effects of NF-κB. It is therefore interesting to speculate that EBNA1 may modulate the ability of LMP1 to activate specific aspects of NF-κB signaling and that this in turn may impact upon the pathogenesis of NPC and other EBV-related tumours. This clearly warrants further investigation.

## Conclusions

Our findings suggest that EBNA1 may play a role in the inhibition of p65 NF-κB in NPC and that this could contribute to NPC pathogenesis by inducing tissue hyperplasia. Viruses have evolved an array of mechanisms to overcome the induction of NF-κB as a way of evading the innate immune response [34,41-43]. We therefore propose that inhibition of canonical NF-κB by EBNA1 may not only contribute to the development of tissue hyperplasia but may also play a role in the pathogenesis of NPC via evasion of host immune responses during early EBV infection.

## Competing interests

The authors declare that they have no competing interests.

## Authors' contributions

RV participated in the design and interpretation of the study, carried out the majority of the experimental work and helped to draft the manuscript. CWD participated in the design and interpretation of the study and helped to draft the manuscript. CH performed immunohistochemical staining and tumour diagnosis. KMS assisted with carrying out and interpreting EMSAs and helped to draft the manuscript. TJO carried out RT-PCR, assisted with the generation of supplementary data and helped to draft the manuscript. KLD carried out RT-PCR, assisted in the validation of lentivirus expression vectors and helped to draft the manuscript. SPM generated the panel of lentivirus vectors and assisted in their validation. JS provided material for immunohistochemistry and carried out tumour diagnosis. JRA participated in the design and interpretation of the study and helped to draft the manuscript. LSY participated in the design and interpretation of the study and helped to draft the manuscript. JDO participated in the design and interpretation of the study, carried out TransAM analysis and mutant EBNA1 experiments and was responsible for drafting the manuscript. All authors have read and approved the final manuscript.

## Supplementary Material

Additional file 1 **Figure S1: EBNA1 does not bind with or relocalise IKKβ to the nucleus in Ad/AH cells**. A PowerPoint file demonstrating that EBNA1 does not bind with or relocalise IKKβ to the nucleus in Ad/AH cells. (A) Pseudo-wild type EBNA1 (with deleted Gly/Ala repeat region) fused to the HaloTag protein (N-terminal) in the pFC14K-CMV backbone plasmid (Halo-EBNA1) (Promega UK, to be described elsewhere) was transiently transfected into Ad/AH cells. Following pull-down of Halo-EBNA1 using affinity resin samples were washed following the manufactures instructions and subjected to immunoblotting for EBNA1, the known cellular EBNA1 binding protein USP7 and IKKβ. Input = whole protein lysate prior to Halo-EBNA1 pull-down, BL = pull-down resin blocked with the supplied blocking ligand, No BL = Halo-EBNA1 pull-down using the supplied resin without the use of the blocking ligand. (B) Immunoblotting for IKKβ was carried out on nuclear and cytosolic extracts from Ad/AH cells stably expressing either EBNA1 or a neomycin control vector (neo). Immunoblotting for SP1 and tubulin was carried out to demonstrate adequate fractionation of nuclear and cytosolic extracts, respectively. Click here for file
