# Supplementary figures and images for: Epstein-Barr virus-encoded EBNA1 inhibits the canonical NF-κB pathway in carcinoma cells by inhibiting IKK phosphorylation
Source: Mol Cancer. 2010 Jan 5;9:1. doi: 10.1186/1476-4598-9-1 (PMC2818691; doi:10.1186/1476-4598-9-1)

## Slide 1
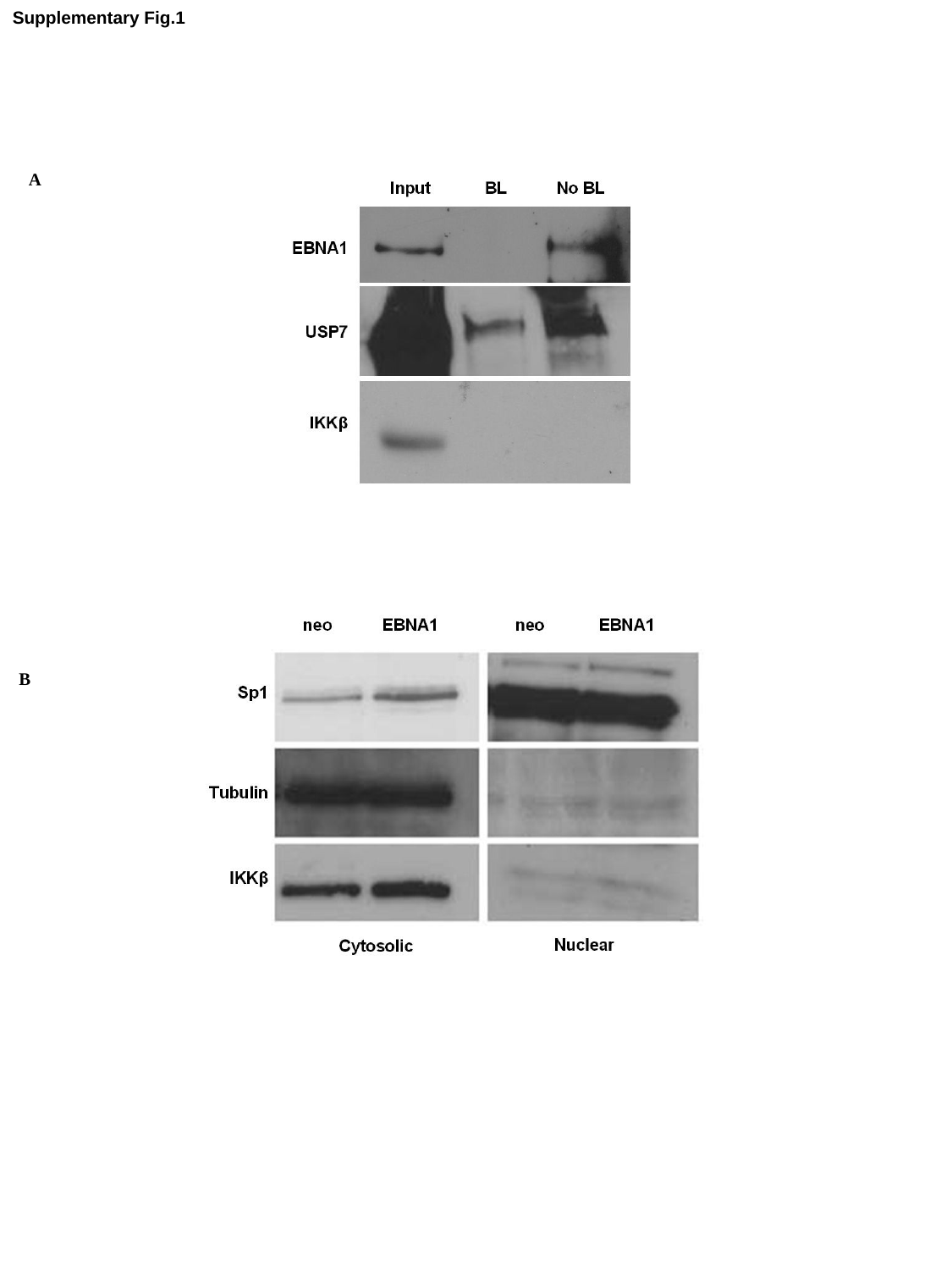

Supplementary Fig.1
A
B

Supplement: Additional file 1 — Figure S1: EBNA1 does not bind with or relocalise IKKβ to the nucleus in Ad/AH cells. A PowerPoint file demonstrating that EBNA1 does not bind with or relocalise IKKβ to the nucleus in Ad/AH cells. (A) Pseudo-wild type EBNA1 (with deleted Gly/Ala repeat region) fused to the HaloTag protein (N-terminal) in the pFC14K-CMV backbone plasmid (Halo-EBNA1) (Promega UK, to be described elsewhere) was transiently transfected into Ad/AH cells. Following pull-down of Halo-EBNA1 using affinity resin samples were washed following the manufactures instructions and subjected to immunoblotting for EBNA1, the known cellular EBNA1 binding protein USP7 and IKKβ. Input = whole protein lysate prior to Halo-EBNA1 pull-down, BL = pull-down resin blocked with the supplied blocking ligand, No BL = Halo-EBNA1 pull-down using the supplied resin without the use of the blocking ligand. (B) Immunoblotting for IKKβ was carried out on nuclear and cytosolic extracts from Ad/AH cells stably expressing either EBNA1 or a neomycin control vector (neo). Immunoblotting for SP1 and tubulin was carried out to demonstrate adequate fractionation of nuclear and cytosolic extracts, respectively. [file 1476-4598-9-1-S1.PPT]
